# A small molecule inhibitor of Nicotinamide N-methyltransferase for the treatment of metabolic disorders

**DOI:** 10.1038/s41598-018-22081-7

**Published:** 2018-02-26

**Authors:** Aimo Kannt, Sridharan Rajagopal, Sanjay Venkatachalapathi Kadnur, Juluri Suresh, Ravi Kanth Bhamidipati, Srinivasan Swaminathan, Mahanandeesha Siddappa Hallur, Rajendra Kristam, Ralf Elvert, Jörg Czech, Anja Pfenninger, Christine Rudolph, Herman Schreuder, Devaraj Venkatapura Chandrasekar, Vishal Subhash Mane, Swarnakumari Birudukota, Shama Shaik, Bharat Ravindra Zope, Raghunadha Reddy Burri, Niranjan Naranapura Anand, Manish Kumar Thakur, Manvi Singh, Reejuana Parveen, Saravanan Kandan, Ramesh Mullangi, Takeshi Yura, Ramachandraiah Gosu, Sven Ruf, Saravanakumar Dhakshinamoorthy

**Affiliations:** 1 Sanofi Research and Development, Industriepark Hoechst, H823, D-65926 Frankfurt am Main, Germany; 2 Jubilant Biosys Ltd, Bangalore, 560022 India; 3 Institute of Experimental Pharmacology, Medical Faculty Mannheim, University of Heidelberg, D-68167 Mannheim, Germany

## Abstract

Nicotinamide N-methyltransferase (NNMT) is a cytosolic enzyme that catalyzes the transfer of a methyl group from the co-factor S-adenosyl-L-methionine (SAM) onto the substrate, nicotinamide (NA) to form 1-methyl-nicotinamide (MNA). Higher NNMT expression and MNA concentrations have been associated with obesity and type-2 diabetes. Here we report a small molecule analog of NA, JBSNF-000088, that inhibits NNMT activity, reduces MNA levels and drives insulin sensitization, glucose modulation and body weight reduction in animal models of metabolic disease. In mice with high fat diet (HFD)-induced obesity, JBSNF-000088 treatment caused a reduction in body weight, improved insulin sensitivity and normalized glucose tolerance to the level of lean control mice. These effects were not seen in NNMT knockout mice on HFD, confirming specificity of JBSNF-000088. The compound also improved glucose handling in ob/ob and db/db mice albeit to a lesser extent and in the absence of weight loss. Co-crystal structure analysis revealed the presence of the N-methylated product of JBSNF-000088 bound to the NNMT protein. The N-methylated product was also detected in the plasma of mice treated with JBSNF-000088. Hence, JBSNF-000088 may act as a slow-turnover substrate analog, driving the observed metabolic benefits.

## Introduction

Nicotinamide N-methyltransferase (NNMT) is a cytosolic enzyme that catalyzes the N-methylation of nicotinamide by transferring a methyl group from S-adenosyl-L-methionine (SAM) to nicotinamide (NA) resulting in the formation of 1-methyl-nicotinamide (MNA) and S-adenosyl-L-homocysteine (SAH)^1,2^. NNMT is predominantly expressed in the liver and in adipose tissue but is also found in other tissues such as kidney, lung, muscle, heart, brain and tumor cells. NNMT is implicated in various disease conditions such as metabolic disorders, neurodegenerative diseases and cancer, and tissue NNMT expression or plasma levels of its product MNA have been proposed as biomarkers for these conditions^3–5^.

Recently, several reports have indicated a role of NNMT in obesity, insulin resistance and type 2 diabetes (T2D)^4,6,7^. NNMT expression and activity were found to be high in white adipose tissue (WAT) samples of mouse models of obesity and insulin resistance (ob/ob, db/db and diet-induced obese (DIO) models)^6,8^. Also, higher NNMT expression was detected in isolated adipocytes from obese as compared to non-obese Pima Indians^9^. Population studies indicate that MNA levels strongly correlate with obesity and diabetes in Chinese individuals^3^. Additionally, urinary MNA levels were found to be elevated in humans with T2D, and in diabetic animals (db/db, obese Zucker rats)^10^. Interventions to improve insulin sensitivity such as exercise and bariatric surgery were shown to decrease NNMT expression in adipose tissue and result in reduction of plasma MNA levels^4^. Animals treated with an antisense oligonucleotide targeting NNMT have been reported to have reduced fat mass, body weight and increased energy expenditure^6^. Further, the role of NNMT in the regulation of hepatic glucose, lipid and cholesterol metabolism was demonstrated using cellular and animal models with adenoviral mediated NNMT knockdown^11^. The identification and characterization of small-molecule NNMT inhibitors has recently been reported^12,13^. However, till date there are no reports on the feasibility of using small molecule modulators of NNMT in preclinical animal models of metabolic disease to validate NNMT as a pharmacological drug target.

The objective of the current study was to identify selective small molecule modulators of NNMT with drug like properties and to test them in preclinical animal models of obesity, insulin resistance and diabetes to provide the first proof of concept for a small molecule targeting NNMT for treating metabolic disorders. A high throughput screening (HTS) campaign was undertaken which eventually led to the identification of a lead compound JBSNF-000088. JBSNF-000088 was co-crystallized with human and mouse NNMT protein and tested in various animal models of diabetes. We demonstrate the pharmacological benefits of a small molecule modulator of NNMT for the first time and explain its mechanism of action.

## Results

### Identification of a small molecule NNMT inhibitor

A chemical library containing > 1 million small molecule compounds was screened for NNMT inhibitors, resulting in the identification of several structurally distinct hit series. The hits were clustered and after several rounds of refinement, JBSNF-000088 (6-methoxynicotinamide), which was found to be active in both human and mouse NNMT enzymes and was taken for further studies. The compound is part of a series of related molecules. The structure-activity relationship within the series have been described elsewhere^14^.

### ***In-vitro*** profile of JBSNF-000088

**Figure 1:**
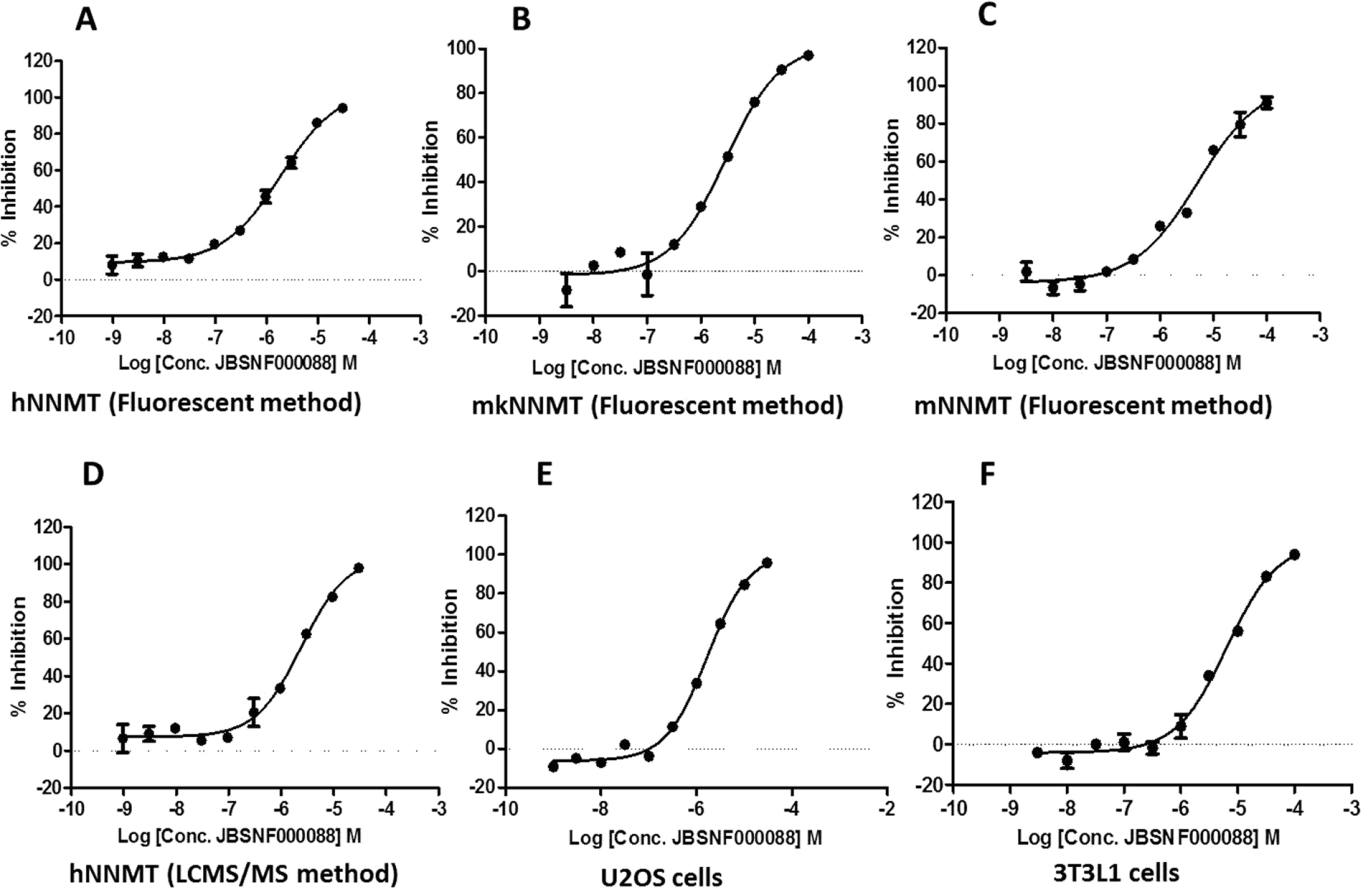
Profile of compound 1 (JBSNF-000088) in enzymatic and cell based assays. JBSNF-000088 inhibits human, mouse and monkey NNMT enzymatic activities in biochemical assay (**A**,**B**,**C**). Inhibition of human NNMT enzyme was demonstrated using LCMS/MS based enzymatic assay (**D**). Cell based assays were carried out using U2OS cells (**E**) and differentiated 3T3L1 cells (**F**).

The concentration-dependent inhibitory effect of JBSNF-000088 on NNMT enzymatic activity was tested using a fluorescence based biochemical assay. The results are shown in Fig. 1. JBSNF-000088 inhibited human NNMT (hNNMT), monkey NNMT (mkNNMT), and mouse NNMT (mNNMT) enzymatic activities with IC_50_ values of 1.8, 2.8, and 5.0 µM, respectively (Fig 1A–C). The activity of JBSNF-000088 against hNNMT was also confirmed by an alternative LCMS/MS detection method with an IC_50_ of 2.4 µM (Fig. 1D). To investigate the inhibitory effect of JBSNF-000088 on endogenous NNMT activity in cells, U2OS or differentiated 3T3L1 cells were treated with JBSNF-000088 for 24 h, and the levels of MNA were assessed by LC-MS/MS. The calculated IC_50_ values are 1.6 and 6.3 µM, respectively (Fig. 1E-F). The approximately 4-fold right shift in potency observed with the compound tested in 3T3L1 cells as compared to U2OS cells may reflect the higher potency on the human enzyme compared to the mouse enzyme as U2OS is a human cell line whereas 3T3-L1 cells are of murine origin. Cytotoxicity of JBSNF-000088 was investigated in HepG2 cells. The cells were incubated with JBSNF-000088 at 10, 30 and 100 µM concentrations for 72 h and cytotoxicity was measured using the CellTiter-Glo assay kit. JBSNF-000088 did not show any toxicity at the tested concentrations (data not shown). The compound was also tested in a hERG assay (patch clamp) and a NaV1.5 assay at 30 µM. No liability was found (<30% inhibition @ 10 µM) (data not shown).

### Co-crystal structures of human and mouse NNMT with JBSNF-000088

A thermal shift assay was performed to assess the binding of JBSNF-000088 to hNNMT and mNNMT prior to co-crystallization studies. JBSNF-000088 was found to be tightly bound to human NNMT in presence of SAM (molar ratio of enzyme:SAM:compound ~1:5:10) with a 5 K shift in melting temperature (Tm). Similar results were obtained with mouse NNMT when the experiment was repeated at higher concentrations of SAM or compound i.e. molar ratio of enzyme: SAM: compound ~1:10:20 (data not shown). Based on these results, crystallization was carried out in the presence of JBSNF-000088 and SAM at molar ratios of enzyme:SAM:compound ~1:5:10 for human NNMT & at molar ratio of enzyme:SAM:compound ~1:10:20 for mouse NNMT. Co-crystals were obtained with JBSNF-000088 for both human and mouse NNMT enzymes and X-ray crystal structures were determined at 2.48 Å and 1.99 Å resolution in P2_1_ and P2_1_2_1_2_1_ space group, respectively. Data collection and refinement statistics are summarized in supplementary Table 1.

**Figure 2:**
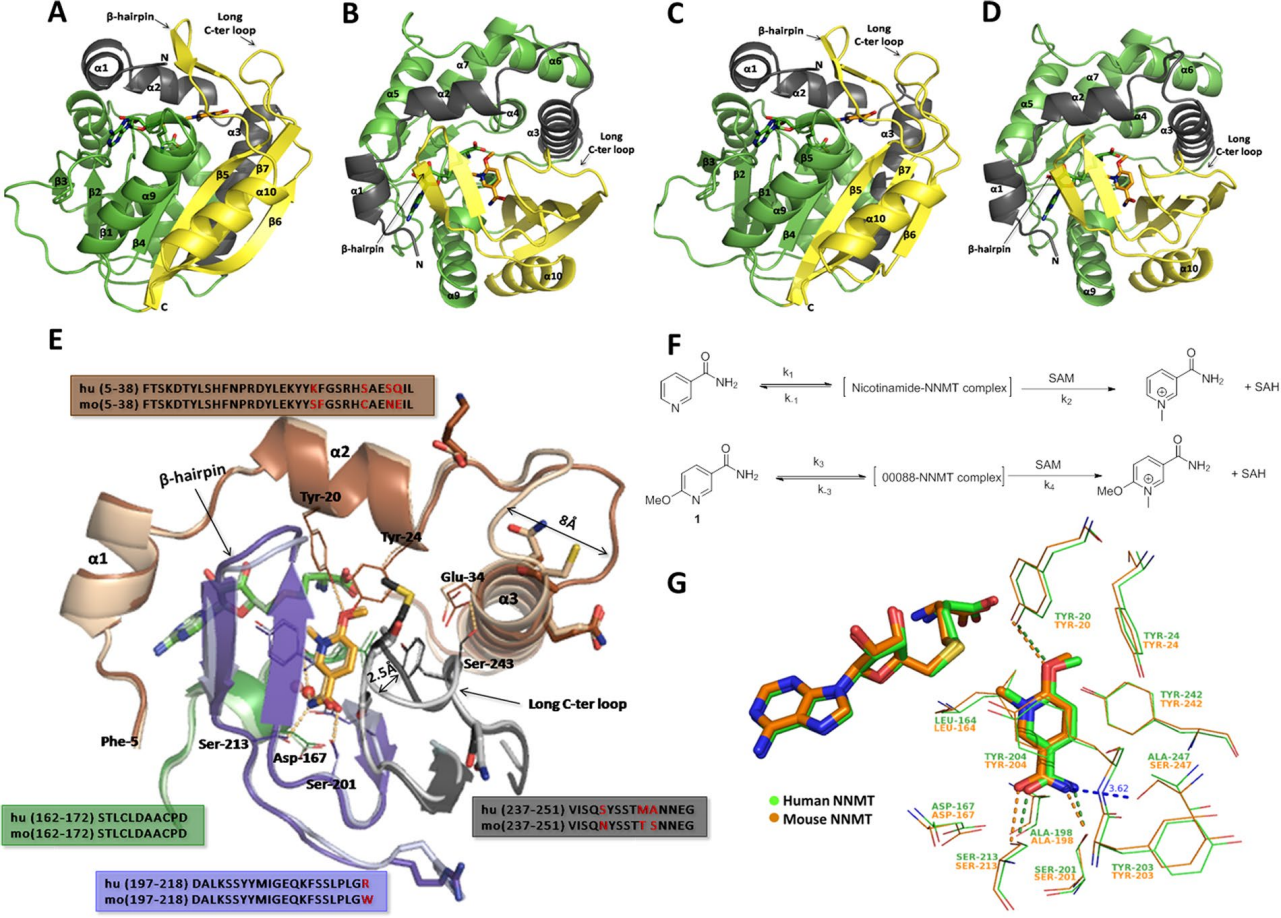
Structures of human and mouse NNMT in complex with SAH and N-methylated JBSNF-00088. **(A**,**B)** Structure of human NNMT in 2 orientations and perpendicular to each other. **(C**,**D)** Structure of mouse NNMT in 2 orientations and perpendicular to each other.N-terminal, Central and C-terminal domains are colored in grey, green and yellow respectively. SAH and N-methylated compound 1 (compound 40)are shown in sticks.**(E)** Overlay of active site region of ternary complex structures of human NNMT and mouse NNMT shown with key structural elements. Human NNMT in dark colored and mouse NNMT in pale colored. Amino acids surrounding N-methylated JBSNF-000088 are shown in lines. H-bond is shown in dotted pink line. Waters are shown as spheres. Compound and SAH are shown in sticks. Loop movement in mouse from human structure is highlighted by double-headed arrow. Amino acids corresponding to the displayed structural elements of human/mouse NNMT are given in the box. Non-conserved amino acids are colored red in sequence and shown as sticks in the structure. **(F)** Structural scheme describing the formation of methylated JBSNF-000088. **(G)** Binding site environment of methylated JBSNF-000088

Human and mouse NNMT proteins are very similar in size, consisting both of 264 amino acid residues. The biologically active unit is a monomer. Both share 92% sequence similarity and 86% sequence identity. Their three-dimensional structures are very similar and overlay within 0.4 Å RMS deviations (Figs 2A–D). The difference in amino acid composition is mainly in the long C-terminal loop (in human: ^238^ISQSYSSTMANNEG^251^; in mouse: ^238^ ISQNYSSTTSNNEG^251^) and helix a3 with its preceding loop (hNNMT: ^25^YKFGSRHSAESQILKHLLKNLFKIFCL^51^, mNNMT: ^25^YSFGSRHCAENEILRHLLKNLFKIFCL^51^ (Fig. 2E).

JBSNF-000088 was found to be methylated in the human and mouse NNMT structures with the cofactor SAM correspondingly being demethylated to SAH. The latter was bound in the cradle formed by loops ^141^Cys-Asp-Val-Thr, ^85^Asp-Tyr-Thr, ^63^Gly-Ser-Gly, ^163^Thr-Leu-Cys and ^69^Tyr-Glu originating from the central domain Fig. 2G). The binding environment of the methylated compound is very similar in human and mouse NNMT. The compound is surrounded by Tyr-20 (from N-terminal domain), Leu-164, Asp-167 (from central domain), Ala-198, Ser-201, Tyr-204, Ser-213, Tyr-242, Ala-247 (from C-terminal domain) and SAH within 4.0 Å. The methylated product is bound below the hairpin structural motif at the nicotinamide pocket, and its pyridine ring stacks between Tyr-204 (from hairpin) and Leu-164. The amide moiety is recognized by two Ser residues from the hairpin (Ser-201 and Ser-213). The methoxy group interacts with Tyr-20 (from N-terminal helix). The protons from the pyridine ring interact with the pi-electron cloud of Tyr-242 in an edge-to-face aromatic interaction. The protons of the methoxy group interact with the pi-electron cloud of Tyr-24. In the binding site of mouse NNMT, a further interaction, though probably weaker, is observed between the amino group of the amide and Ser-247. In the human NNMT, this position has an alanine. The methyl group of methylated JBSNF-000088 is in close proximity (~3.4 Å) to the sulfur atom of SAH. Asp-167 (from central domain) at the active site is found stacked against Asp-197 (from C-terminal domain).

### eADME profile and pharmacokinetics of JBSNF-000088

The overall eADME, safety and selectivity profile of JBSNF-000088 is summarized in Table 1. The solubility of JBSNF-000088 in aqueous buffer was found to be >200 µM. At a concentration of 20 µM, the compound did not inhibit any of the tested cytochrome P450 enzymes. It was stable in liver microsomes from different species and in human hepatocytes (>70% remaining after 30 minutes). In addition, the compound was negative in the micronucleus test and the Ames mutagenicity test. When tested at a concentration of 10 µM against a panel of 34 other targets, primarily receptors, transporters and enzymes, the compound did not show agonistic or antagonistic/inhibitory effects on any of these targets. The detailed compound selectivity profile is shown in supplementary Table 2.

**Table 1:** ADME, PK and safety profile of JBSNF-000088.

Parameter	JBSNF-000088
Metabolic stability h/r/m/ microsomes (% remaining)	90/91/79
Solubility (µM)	208
Caco-2 permeability (nm/sec)	252
Caco2 Efflux ratio	1.04
Cyp Inhibition	>20 uM
Cl/Vd/F%-mL/min/kg and L/Kg	20.5/0.69/43
Ames test	negative
Micronucleus test	negative
Selectivity panel	clean^a^

^a^JBSNF-000088 was tested against a panel of 34 receptors, transporters and enzymes. Results are shown in Supplementary Table 2.

**Figure 3:**
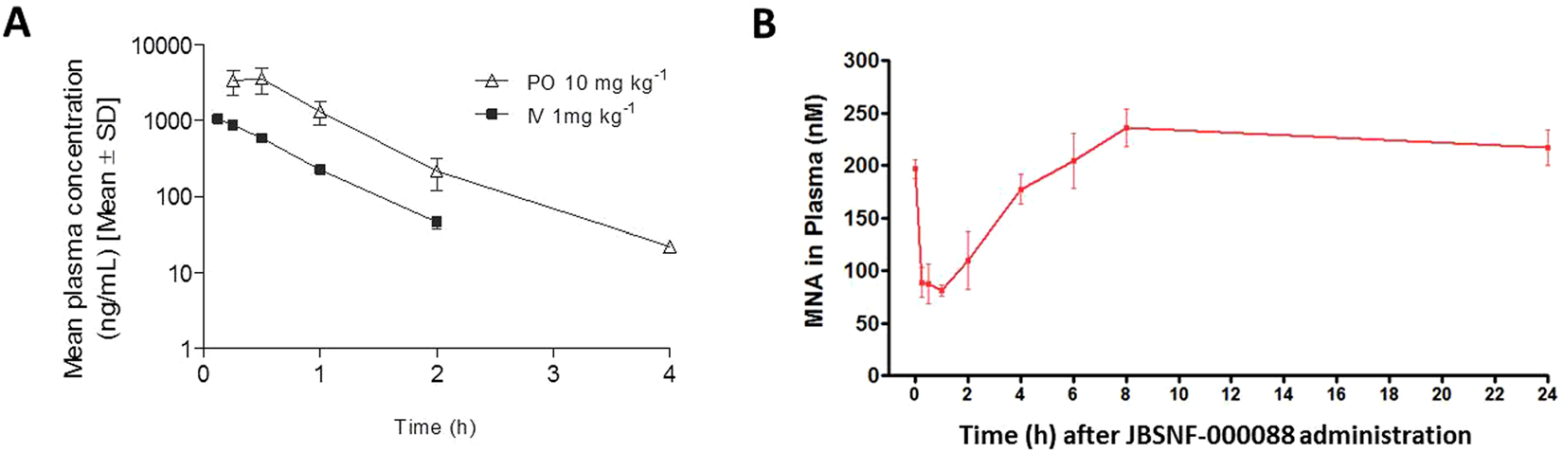
JBSNF-000088: Pharmacokinetics profile and target engagement. (**A**) Plasma concentration versus time profile of JBSNF-000088 in C57BL/6 mice. **(B)** Plasma MNA concentrations from the same study. Data presented as mean ± SEM of 3 animals at each time point.

*In-vivo* pharmacokinetics (PK) studies demonstrated that the compound has good exposure and a moderate half-life (Fig. 3A). Intravenous administration of JBSNF-000088 resulted in low plasma clearance of 21 mL min^−1^ kg^−1^, which is approximately 25% of hepatic blood flow in mice. The volume of distribution at steady state was found to be 0.7 L kg^−1^, which is approximately equal to total body water in mice. However, JBSNF-000088 exhibited a very short plasma half-life of 0.5 h upon intravenous administration. Upon oral gavage, JBSNF-000088 at 10 mg kg^−1^ resulted in a C_max_ of 3568 ng mL^−1^ with a T_max_ value of 0.5 h, indicating rapid absorption in the intestine. The oral bioavailability was found to be approximately 40%. However, similar to intravenous route, the apparent oral half-life was found to be 0.4 h (Fig. 3A). Pharmacodynamics studies investigating the effect of compound treatment on plasma MNA levels were conducted in C57BL/6 mice. Animals were dosed at 50 mg kg^−1^ orally with JBSNF-000088. Significant reductions in plasma MNA levels were observed till 4 h post dosing (Fig. 3B). Plasma exposure of JBSNF-000088 was detectable up to 8 h post dosing.

### JBSNF-000088 reduces body weight and restores glucose tolerance in mice with diet-induced obesity (DIO)

**Figure 4:**
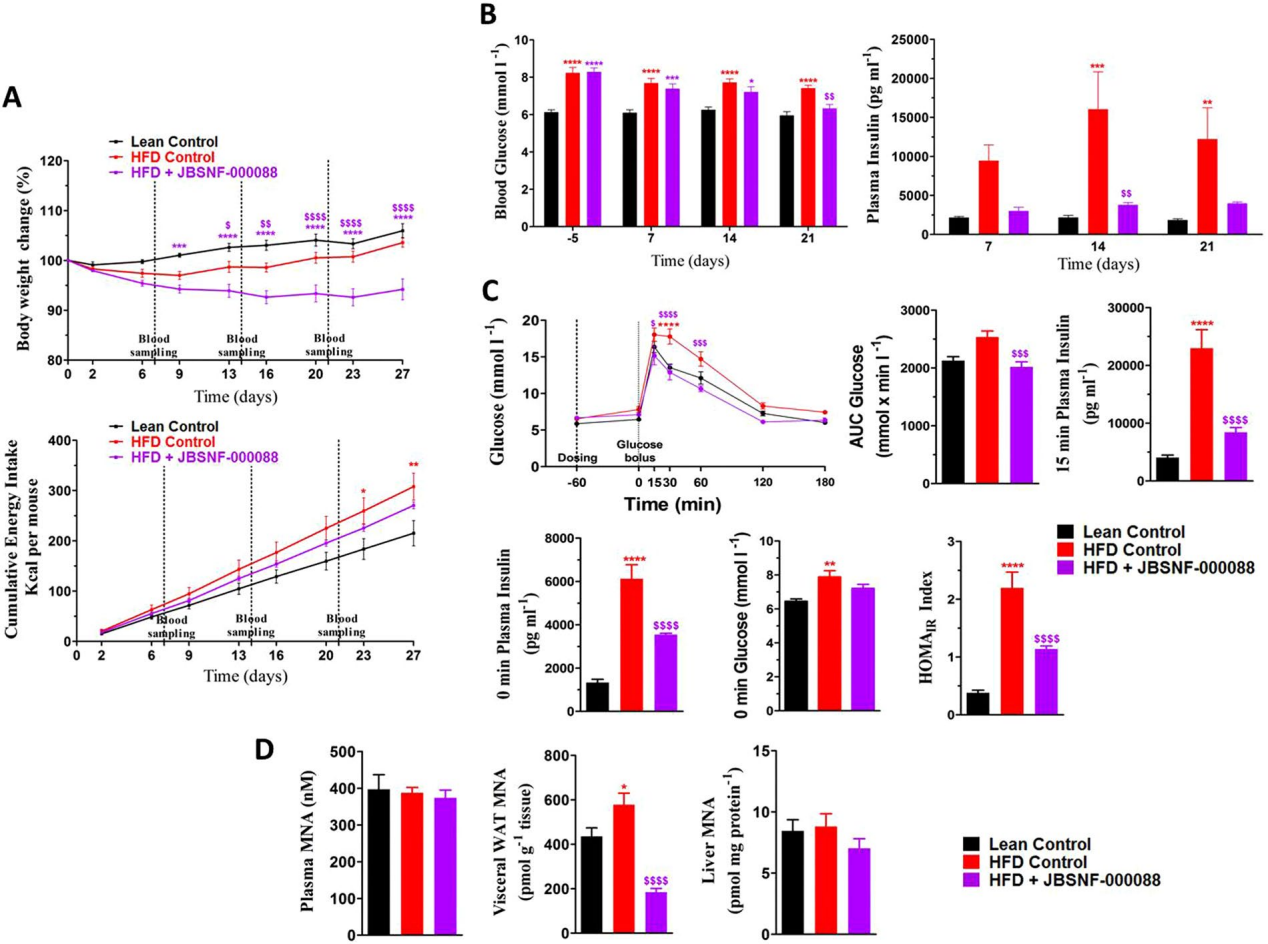
Effect of 4-w treatment with JBSNF-000088 (50 mg kg^−1^ bid) in mice with diet-induced obesity. (**A)** Body weight changes (%) and cumulative energy intake in lean control animals and HFD fed animals treated with vehicle or JBSNF-000088. **(B)** Fed blood glucose and plasma insulin profile of lean control animals and HFD-fed animals treated with vehicle or JBSNF-000088. **(C)** OGTT profile, AUC of blood glucose, 15 min plasma insulin, 0 min plasma insulin, 0 min blood glucose and HOMA-IR index in lean control animals and HFD fed animals treated with vehicle or JBSNF-000088. **(D)** MNA concentrations in plasma, visceral WAT and liver samples of lean control animals and HFD fed animals treated with vehicle or JBSNF-000088. Animals were housed as n = 5 per cage and individual animal body weight, food and water consumption were recorded twice weekly for the duration of the study. Food consumption was expressed as cumulative energy intake. Data presented as mean ± SEM of 8–10 animals. *P < 0.05, **P < 0.01, ***P < 0.001 and ****P < 0.0001 when compared with lean Control and ^$^*P* < 0.05, ^$$^*P* < 0.01, ^$$$^*P* < 0.001 and ^$$$$^*P* < 0.0001 when compared with HFD Control. Two way ANOVA followed by Bonferroni’s post-hoc test (**A**,**B** and **C** (blood glucose during OGTT)); One way ANOVA followed by Bonferroni’s post-hoc test (**C** and **D**).

Based on the good physicochemical properties, pharmacokinetics, and pharmacodynamics leading to sustained reductions in plasma MNA levels, compound JBSNF-000088 was investigated in chronic efficacy studies using diet induced obesity (DIO), ob/ob and db/db mouse models. The compound was tested at 50 mg kg^−1^, b.i.d, via oral route of administration. In the DIO model, the animals were pre-fed with a high-fat diet (HFD) for 14 weeks before treatment with JBSNF-000088 or vehicle for four weeks. Animals remained on HFD throughout the treatment period. At the onset of treatment, animals on HFD weighed 46.6 ± 0.6 grams whereas age-matched control animals on normal rodent chow weighed 34.0 ± 0.7 grams. Throughout the treatment period, the JBSNF-000088 group showed statistically significant reduction in body weight (%) as compared to the vehicle treated group (Fig. 4A). The cumulative food intake was comparable between the JBSNF-000088 treatment group and the vehicle treated group (Fig. 4A). JBSNF-000088 treatment led to a statistically significant reduction in fed blood glucose on day 21 (*p* < 0.01) compared to vehicle control (Fig. 4B). Further, the treatment group also showed a trend to a reduction in fed plasma insulin that was statistically significant on day 14 (*p* < 0.01) compared to HFD control (Fig. 4B). Twice daily oral gavage administration of JBSNF-000088 at 50 mg kg^−1^ to DIO mice led to a statistically significant improvement in oral glucose tolerance on day 28 (Fig. 4C) with glucose tolerance on JBSNF-000088 being normalized to that of the chow control group. Significantly lower AUC blood glucose (*p* < 0.001) was observed in the compound treated group as compared to HFD control. Compound treatment resulted in statistically significant lower plasma insulin (*p* < 0.0001) levels at 0 minutes as well as 15 minutes of OGTT compared to HFD control (Fig. 4C). As a consequence of lower fasting glucose and insulin levels, there was statistically significant improvement in HOMA-IR index (*p* < 0.0001) in JBSNF-000088-treated mice compared to the vehicle-treated HFD controls (Fig. 4C). Plasma and liver MNA levels were not different from those of the vehicle treatment animals as animals were sacrificed following the oGTT and therefore >4 hours after the last compound administration when MNA levels return back to normal (Fig. 3B). However, a statistically significant reduction in MNA levels in visceral WAT (*p < *0.0001) compared to HFD control (Fig. 4D) remained. JBSNF-000088 at 50 mg kg^−1^ b.i.d. led to a statistically significant reduction in plasma triglyceride (*p < *0.001) and liver triglyceride (*p < *0.05) compared to HFD control (data not shown).

### JBSNF-000088 improves glucose handling but not body weight in a genetic model of insulin resistance (ob/ob)

**Figure 5:**
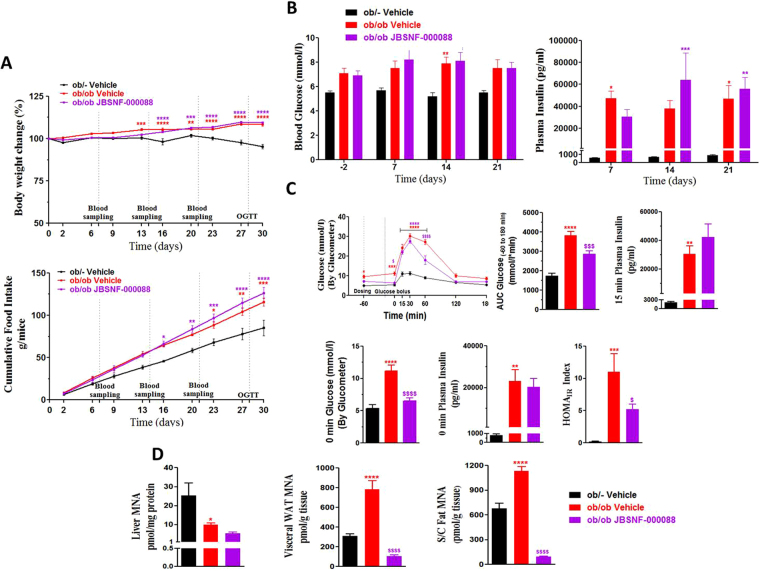
Effect of 4-w treatment with JBSNF-000088 (50 mg kg^−1^ bid) in ob/ob mice. **(A)** Body weight changes (%) and cumulative energy intake in ob/- control animals and ob/ob animals treated with vehicle or JBSNF-000088. **(B)** Fed blood glucose and fed plasma insulin profile of ob/- control animals and ob/ob animals treated with vehicle. **(C)** OGTT profile, AUC of blood glucose, 15 min plasma insulin, 0 min blood glucose, 0 min plasma insulin and HOMA-IR index in ob/- control animals and ob/ob animals treated with vehicle. **(D)** MNA concentrations in liver, visceral WAT and s/c fat samples of ob/- and ob/ob mice treated with vehicle or JBSNF-000088. Animals were housed as n = 5 per cage and individual animal body weight, food and water consumption were recorded twice weekly for the duration of the study. Food consumption was expressed as cumulative energy intake. Data presented as mean ± SEM of 8–10 animals. *P < 0.05, **P < 0.01, ***P < 0.001 and ****P < 0.0001 when compared with ob/- control and ^$^*P* < 0.05, ^$$$^*P* < 0.001 and ^$$$$^*P* < 0.0001 when compared with ob/ob vehicle ontrol. Two way ANOVA followed by Bonferroni’s post-hoc test (**A**,**B** and **C** (blood glucose during OGTT)); One way ANOVA followed by Bonferroni’s post-hoc test (**C** and **D**).

At the onset of the 30-day treatment period with either vehicle or JBSNF-000088 (50 mg/kg b.i.d p.o.), ob/ob mice were 12 weeks old and weighed 54.0 ± 1.0 grams whereas age-matched lean ob/- control animals had an average body weight of 31.3 ± 0.7 grams. Throughout the treatment period, the ob/ob mice in the vehicle control group and the JBSNF-000088 treatment group gained comparable weight and it was more than that of ob/- animals (Fig. 5A). The cumulative food intake was comparable between the JBSNF-000088 treatment group and the vehicle treated group (Fig. 5A). The JBSNF-000088 treatment group showed no reduction in fed blood glucose or insulin on day 21 compared to vehicle control (Fig. 5B). Twice daily oral gavage administration of JBSNF-000088 at 50 mg kg^−1^ to ob/ob mice led to statistically significant improvement in glucose tolerance on day 28 (Fig. 5C), with significantly (*p* < 0.001) lower AUC blood glucose under JBSNF-000088 compared to vehicle control (Fig. 5C). Furthermore, mice treated with JBSNF-000088 at 50 mg kg^−1^ b.i.d. showed a statistically significant improvement (*p < *0.001) in HOMA-IR index compared to vehicle control (Fig. 5C). Of note, improvement in glucose tolerance and HOMA-IR were not accompanied by a reduction in plasma insulin under fed conditions on days 7, 14 or 21 (Fig. 5B), under fasting conditions on day 28 or 15 minutes after the oral glucose bolus (Fig. 5C). As in the DIO study, treatment with JBSNF-000088 for 30 days showed statistically significant reduction in MNA levels in visceral WAT and subcutaneous fat (*p* < 0.0001) but not in liver compared to vehicle control (Fig. 5D).

### JBSNF-000088 moderately improves glucose handling in a genetic model of diabetes (db/db mice)

**Figure 6:**
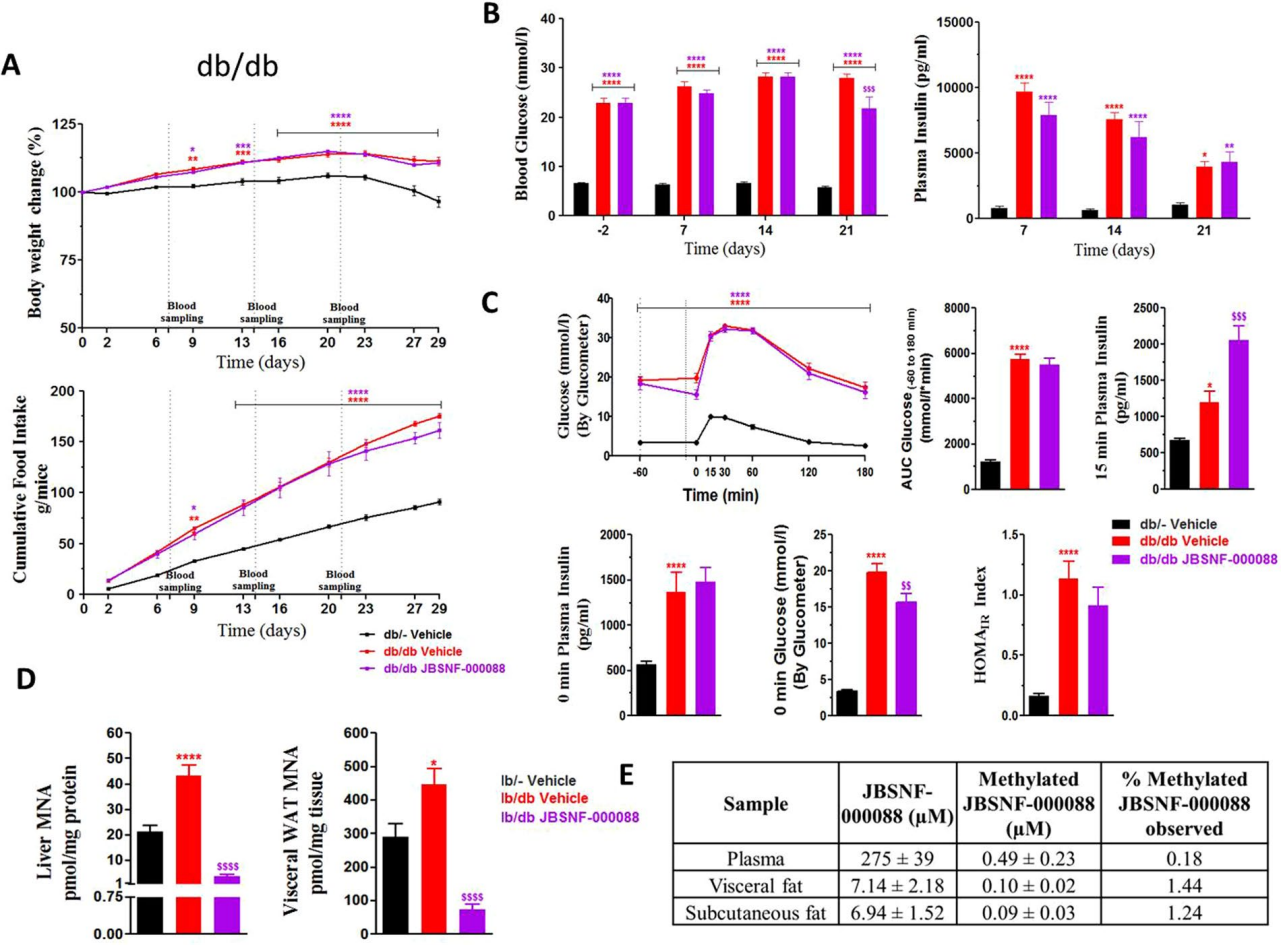
Effect of 4-w treatment with JBSNF-000088 (50 mg kg^−1^ bid) in db/db mice. (**A)** Body weight changes (%) and cumulative energy intake in db/- controls and db/db mice treated with vehicle or JBSNF-000088. **(B)** Fed blood glucose and fed plasma insulin profile of db/- controls and db/db mice treated with vehicle or JBSNF-000088. **(C)** OGTT profile, AUC of blood glucose, 15 min plasma insulin, 0 min plasma insulin, 0 min blood glucose and HOMA-IR index in db/- control mice and db/db mice treated with vehicle or JBSNF-000088. **(D)** MNA concentrations in liver and visceral WAT samples of db/- controls and db/db mice treated with vehicle or JBSNF-000088. **(E)** Estimation of methylated JBSNF-000088 and unmethylated JBSNF-00088 in male db/db mice dosed with unmethylated JBSNF-00088. Animals were housed as n = 5 per cage and individual animal body weight, food and water consumption were recorded twice weekly for the duration of the study. Food consumption was expressed as cumulative energy intake. Data presented as mean ± SEM of 8–10 animals. ^*^P < 0.05, **P < 0.01, ***P < 0.001 and ****P < 0.0001 when compared with db/- control and ^$$^*P* < 0.01, ^$$$^*P* < 0.001 and ^$$$$^*P* < 0.0001 when compared with db/db Control. Two way ANOVA followed by Bonferroni’s post-hoc test (**A**,**B** and **C** (blood glucose during OGTT)); One way ANOVA followed by Bonferroni’s post-hoc test (**C** and **D**).

At the onset of the four-week treatment with either vehicle or JBSNF-000088, db/db mice were 8 weeks of age and had an average body weight of 44.2 ± 0.6 grams, compared to age-matched lean db/- control animals that weighed 28.4 ± 0.3 grams. Throughout the treatment period, the db/db mice in the vehicle control group and the JBSNF-000088 treatment group gained comparable weight which was more than that gained by the db/- animals (Fig. 6A). The cumulative food intake was comparable between the JBSNF-000088 treatment group and the vehicle treated group (Fig. 6A). Unlike ob/ob, the db/db mice were overtly diabetic with fed glucose levels exceeding 20 mmol L^−1^ at the onset of treatment and remained so throughout the treatment period. The JBSNF-000088 treatment group showed a moderate but statistically significant reduction in fed blood glucose on day 21 (*p < *0.001) compared to vehicle control (Fig. 6B). Further, the treatment group showed statistically significant reduction in fed plasma insulin on day 14 (*p* < 0.01) compared to HFD control (Fig. 6B). Upon twice daily oral gavage administration of JBSNF-000088 at 50 mg kg^−1^ to db/db mice there was a trend towards improvement in glucose tolerance on day 26 that was, however, not statistically significant (Fig. 6C). Slightly lower AUC blood glucose was observed in the compound treated group as compared to vehicle control (Fig. 6C). Fed insulin levels on days 7, 14 and 21 (Fig. 6B) and fasting insulin levels before administration of the oral glucose bolus on day 28 (Fig. 6C) were not different between vehicle and JBSNF-00088. Also, there was no change in HOMA-IR upon treatment with the NNMT inhibitor (Fig. 6C). Treatment with JBSNF-000088 for 30 days showed statistically significant reduction in MNA levels in liver and visceral WAT and s/c fat (*p < *0.0001) compared to vehicle control (Fig. 6D). We also attempted to see if we could detect the methylated product of JBSNF-000088 in the plasma and various tissues. The data is shown in Fig. 6E. The samples from db/db mice study clearly showed the formation of methylated JBSNF-000088 upon oral administration of JBSNF-000088. The methylated compound could be detected in plasma and adipose tissue (Fig. 6E).

**Figure 7:**
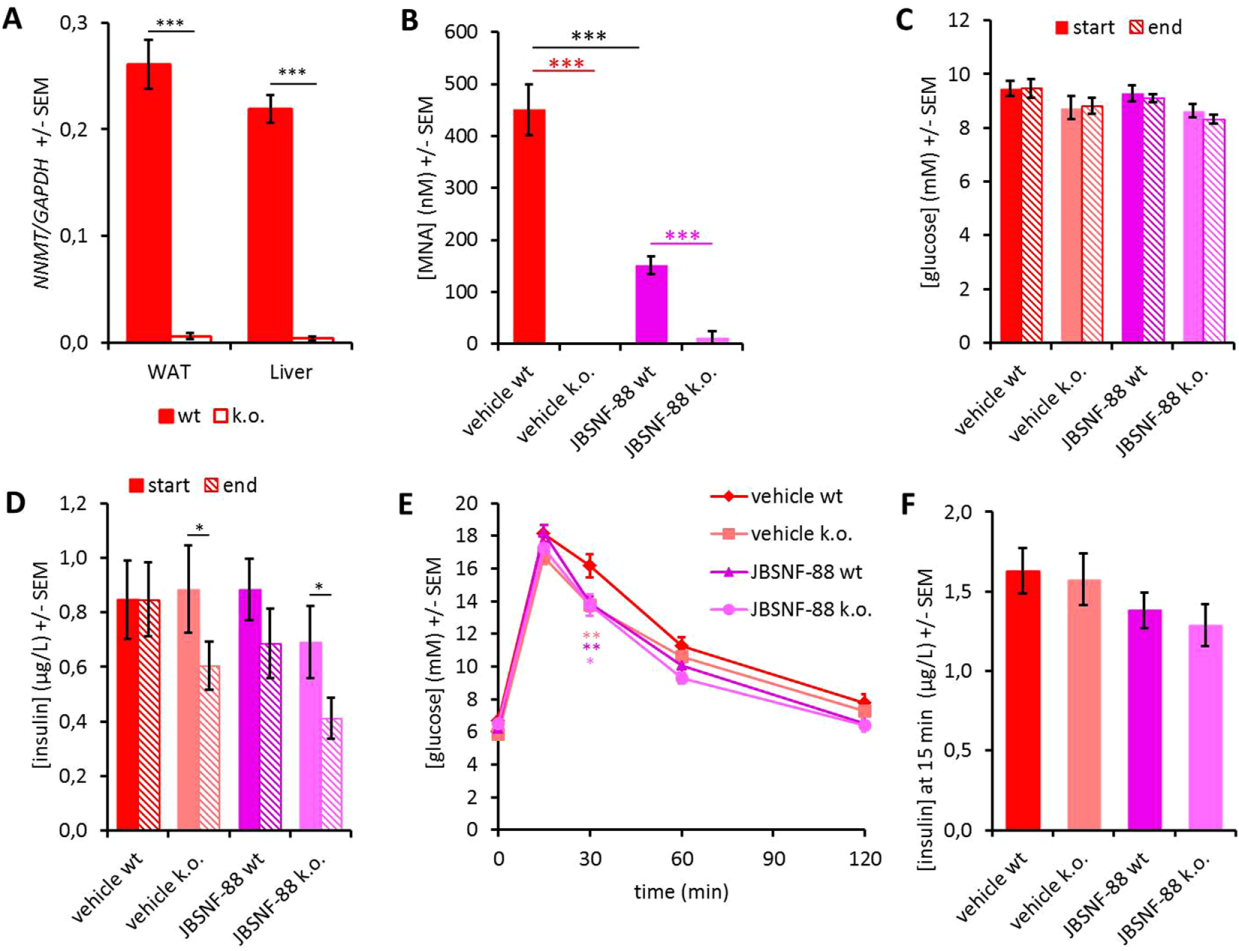
Effect of 4-w treatment with JBSNF-000088 (50 mg kg^−1^ bid) in NNMT knockout animals on HFD. **(A)** *NNMT* expression in white adipose tissue and liver of wild-type and NNMT knockout mice. **(B)** Plasma MNA concentrations in wild-type or knockout animals with or without treatment with JBSNF-000088. **(C)** Plasma glucose concentrations under fed conditions before (filled columns) and after (hatched columns) four weeks of treatment with JBSNF-000088. **(D)** Plasma insulin concentrations before and after four weeks of treatment with JBSNF-000088. **(E)** Oral glucose tolerance test after four weeks of treatment with JBSNF-000088. **(F)** Plasma insulin levels 15 min after oral glucose bolus in the glucose tolerance test. *p < 0.05, **p < 0.01; ***p < 0.001 vs the other indicated column.

To determine whether the observed effects of JBSNF-000088 on insulin sensitivity and glucose tolerance are specific to NNMT inhibition, the compound was also tested in whole-body NNMT knockout mice that were generated by crossing NNMT fl/fl with ZP3-cre mice. Figure 7A shows that animals are devoid of NNMT expression in liver and white adipose tissue. Both knockout animals and wild-type litter mates were put on HFD for 18 weeks and then treated with vehicle of JBSNF-000088 (50 mg/kg b.i.d. p.o.) for four weeks. Body weight at onset of treatment was 32.2 ± 1.3 gram for wild-type and 30.7 ± 1.2 gram for the knockout mice. Compound JBSNF-00088 at 50 mg kg^−1^ b.i.d was well tolerated over four weeks with no change in food intake in either wild-type or knockout animals. At the end of the 28-day treatment period, plasma MNA levels one hour after the last compound administration were determined: No plasma MNA was detectable in the knockout animals confirming that NNMT is exclusively responsible for nicotinamide methylation. Upon treatment of wild-type animals with JBSNF-000088, there was a significant reduction in plasma MNA demonstrating target engagement by the compound (Fig. 7B). Fed glucose levels after 28 days of treatment were not different between wild-type or knockout mice or between vehicle or JBSNF-000088 treatment (Fig. 7C). There was a moderate but significant decrease in fed insulin in the knockout animals that was not observed in wild type. In the latter, a trend to reduced insulin levels was seen after four weeks of treatment with JBSNF-000088 that was, however, not statistically significant (Fig. 7D). Of note, there was an improvement in glucose tolerance observed in both the knockout and the JBSNF-000088 treated wild-type mice compared to the respective vehicle treated animals (Fig. 7E). Importantly, there were no differences in insulin level or glucose-handling in *NNMT* knockout mice treated with JBSNF-000088 compared to vehicle-treated *NNMT* knockout mice. This lack of effect in the knockout, together with the clean *in-vitro* profile (supplementary Table 2) provides further support for the metabolic benefits of JBSNF-000088 being specifically mediated by NNMT inhibition.

## Discussion

NNMT has been proposed as a pharmacological target for treating obesity and type 2 diabetes based on higher NNMT expression and plasma MNA levels in individuals with obesity or T2D and animal studies using antisense-oligonucleotide mediated knockdown of NNMT^3,4,6^. Here we report a small molecule, JBSNF-000088 which is a substrate analog of nicotinamide, that inhibits NNMT activity across species, reduces plasma MNA levels by ~50% and drives insulin sensitization, glucose metabolism and body weight reduction in DIO mice. In mice with deficient leptin signaling, no weight loss and only moderate improvements in glucose handling were observed upon NNMT inhibition *(vide infra)*.

JBSNF-000088 possesses good physico-chemical, pharmacokinetic properties and did not have any obvious safety or drug-drug interaction liabilities. The reduction in glucose and improvements in insulin sensitivity in DIO mice correlated well with the exposure levels of JBSNF-000088 and reduction of MNA levels in plasma, visceral WAT and subcutaneous fat.

In mice with high fat diet-induced obesity and insulin resistance, treatment with JBSNF-000088 prevented further weight gain, reduced hyperglycemia and normalized glucose tolerance to that of the lean control animals. These results are in good agreement with the reduction in weight gain and improvement in insulin sensitivity observed in mice on high-fat diet treated with an NNMT antisense oligonucleotide^6^. Of note, with a body weight of 47 grams the mice in our study were already obese at the initiation of compound treatment (intervention mode) whereas in the antisense study treatment started early, at a body weight of approximately 25 grams (prevention mode). Body weight loss relative to vehicle-treated control mice occurred despite similar food intake. A likely explanation for this effect is an increase in energy expenditure rather reduced feeding efficiency as noted by Kraus *et al*.^6^ who observed an increase in oxygen consumption but unchanged faecal lipid excretion in mice treated with an NNMT antisense oligonucleotide.

A reduction in plasma insulin was seen already after seven days of treatment with JBSNF-000088 in DIO mice, at a time when there was little difference in body weight between compound-treated and vehicle control animals (Fig. 4b). It is tempting to suggest that this indicates a weight-independent improvement in insulin sensitivity upon NNMT inhibition. However, reduction in glucose levels were only noted at later time points, and in the absence of a weight-matched control group it cannot be ruled out that improvement in glucose handling is predominantly secondary to weight loss.

The effect of JBSNF-000088 treatment and glucose and weight homeostasis was much less pronounced in genetic models of insulin resistance and diabetes. In both ob/ob and db/db mice, there was no influence of JBSNF-000088 on body weight. An improvement in glucose tolerance was seen in ob/ob and a reduction in fed blood glucose observed in db/db mice upon chronic NNMT inhibition. However, these effects were comparably moderate. The likely reason for this is pronounced hyperphagia in both models that is predominantly driven by the lack of leptin signaling, and the resulting massive obesity that drives the development of insulin resistance and diabetes. Apparently, the effect of NNMT inhibition is not strong enough to override this mechanism. Such lack of efficacy has also been noticed for other anti-obesity or anti-diabetic drugs like the GLP1-receptor agonist liraglutide that have no effect on body weight and moderate influence on glucose homeostasis in db/db mice upon two weeks of treatment at 100 µg/kg bid^15,16^. A more thorough investigation of the dependence of the metabolic effects of NNMT inhibition on active leptin signaling may be possible by performing leptin supplementation studies in ob/ob animals that were, however, beyond the scope of the work described here.

Studies using wild type and NNMT knockout mice placed on a high fat diet supported the selectivity of JBSNF-000088 for NNMT. The intention of these studies was to demonstrate that JBSNF-000088 did not cause any further metabolic benefit in NNMT knockout animals compared to vehicle-treated NNMT knockout mice, and that the magnitude of metabolic changes upon administration of JBSNF-000088 to wild-type mice does not go beyond that of the NNMT knockout. Indeed, treatment with JBSNF-000088 resulted in improvements in glucose handling in WT mice whereas in NNMT knockout mice no difference between JBSNF-00008-treated and vehicle-treated controls was observed. Of note, weight gain on high-fat diet was limited for both wild-type and knockout animals. After 18 weeks of HFD, animals only weighed 32.2 grams (wild type) or 30.7 grams (knockout animals). The reason for this lack of weight gain is unknown. Correspondingly, the mice were not overtly insulin-resistant or glucose-intolerant, and overall differences in glucose handling between knockout and wild-type animals were comparatively small. Yet we could demonstrate reductions in fed insulin and an improvement in glucose upon treatment with JBSNF-000088 to the level of the knockout animals (Fig. 7C and D). Notably, no further improvement was seen when the knockout animals were treated with the NNMT inhibitor.

The exact mechanism by which NNMT activity/MNA levels trigger the downstream signaling leading to insulin resistance is yet to be fully understood^11^. Several possibilities have been proposed, including pathways involving reactive oxygen species production^17,18^, modulation of NAD + / NADH ratio/levels^6^, a decrease in cellular methylation potential due to changes in SAM/SAH ratio^6,19^, increased polyamine flux^6^ or reduced NAD^+^ availability^18,20,21^ although the latter has been discussed as unlikely because of the high Km value of NNMT for nicotinamide compared to NAMPT, the rate-limiting enzyme of the salvage pathway for NAD^+^ synthesis^1^. An influence of NNMT on sirtuin activity has been described that is not mediated by limited NAD^+^ availability, but by stabilization of Sirt1 protein through inhibition of its ubiquitination^11^.

Our attempts to determine the binding mode of JBSNF-000088 using mouse and human NNMT proteins by X-ray crystallography led to the finding that JBSNF-000088 is methylated and the methylated form of JBSNF-000088 is bound to the active substrate binding site in both proteins with the co-factor SAM turning into the demethylated form (SAH). This supports the hypothesis that JBSNF-000088 may act as a substrate analog, competing with the natural substrate NA and inhibiting the binding of NA to the active site of the enzyme. Once bound to the active site, transfer of methyl group from SAM can lead to the formation of N-methylated JBSNF-000088. Interestingly, the N-methylated product was also found in the circulating plasma of animals dosed with the un-methylated small molecule (JBSNF-000088), though only to a small extent. The latter may be explained by JBSNF-000088 being a potent binder but poor substrate for NNMT with resulting slow turnover. However, in the NNMT co-crystal, due to the high enzyme concentration, formation of the N-methylated compound was clearly detectable. The N-methylated product of JBSNF-000088 itself is a poor inhibitor of NNMT, with an IC_50_ value of > 30 µM (20% inhibition at 30 µM). Therefore, JBSNF-000088 may act as a competitive substrate analog that is very slowly converted to its N-methylated product.

To our knowledge, this is the first proof-of-concept study using a small molecule modulator of NNMT in animal models of metabolic disease to demonstrate pharmacological benefits. Our study opens up the possibility of developing small molecule modulators of NNMT to test in patients with metabolic disorders.

## Methods

All experiments were performed in accordance with relevant guidelines and regulations

### Synthesis of 6-methoxynicotinamide-JBSNF-000088

**Figure 8:**
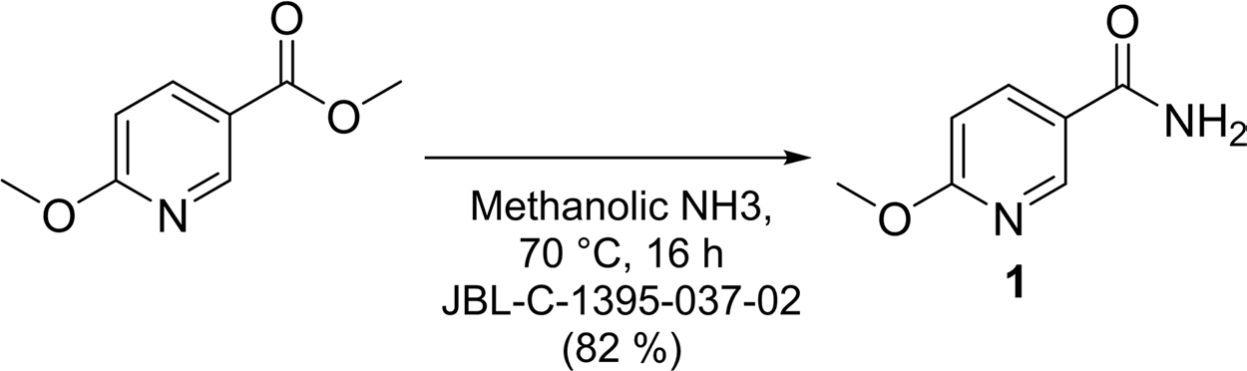
Reaction scheme for synthesis of JBSNF-000088 (compound 1) from methyl 6-methoxynicotinate

As depicted in Fig. 8, a solution of methyl 6-methoxynicotinate (20 g, 119.64 mmol) in methanolic ammonia (200.0 mL) was stirred for 16 h at 70 °C. After completion of the reaction [monitored by TLC, 5% MeOH-DCM], reaction mixture was concentrated under reduced pressure to get desired crude compound. The obtained crude was triturated with diethyl ether (100 mL) and filtered through Buchner funnel, dried under vacuum to afford the desired product as off white solid (15.0 g, 82%). ^1^H NMR (DMSO-d_6_, 400 MHz) δ 3.88 (s, 3 H), 6.84 (d, J = 8.8 Hz, 1 H), 7.34 (s, 1 H), 7.92 (s, 1 H), 8.10 (dd, J = 8.4 Hz, J = 8.4 Hz, 1 H), 8.66 (d, J = 1.6 Hz, 1 H); MS (ESI) m/z 153.1 (M + H) + ; HPLC Purity @ 254 nm, 99.71%.

### Synthesis of -carbamoyl-2-methoxy-1-methylpyridin-1-ium trifluoromethanesulfonate JBSNF-000567 (N-methylated JBSNF-000088)

**Figure 9:**
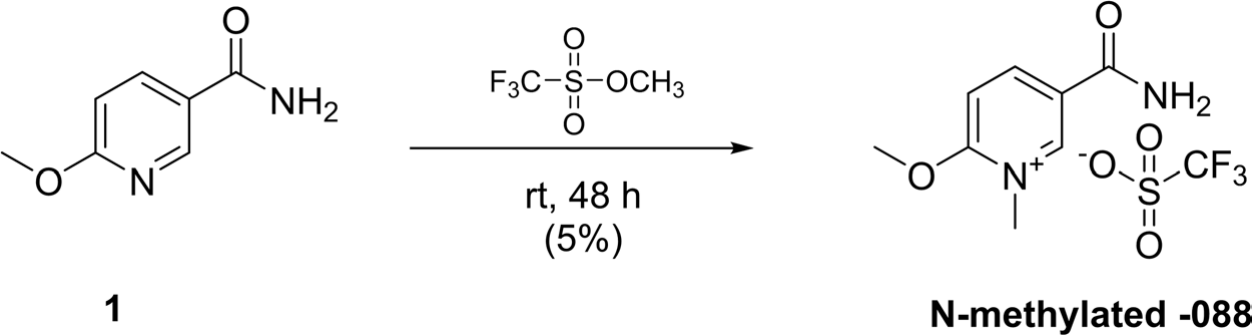
Reaction scheme for N-methylation of compound JBSNF-000088.

As shown in Figs. 9, 6-methoxynicotinamide (0.1 g, 0.65 mmol) and methyl trifluoromethanesulfonate (0.7 mL) was taken a CEM Vial at room temperature. The mixture was stirred for 48 h at room temperature. The mixture was concentrated under reduced pressure to get crude compound. The obtained crude was purified by Prep HPLC using CHIRALPAK IA (250 mm × 4.6 mm × 5μm) and Methanol (Plain without additive)(100%) as Mobile phase with Flow rate 0.5 mL/min. to afford the title compound (0.01 g, 5%). ^1^H NMR (DMSO-d_6_, 400 MHz) δ 3.82 (s, 3 H), 3.91(s,3 H), 6.917 (d, J = 8.8 Hz, 1 H), 8.16–8.13 (m,1 H), 8.37 (d, J = 2.0 Hz, 1 H); MS (ESI) m/z 168.2 (M + H) + ; HPLC Purity @254 nm 99.75%.

### Fluorescence based NNMT enzymatic assay

NNMT activity was measured with the fluorescence enzymatic assay^22,23^. MNA formed in the NNMT reaction reacts with acetophenone in the presence of KOH and formic acid and forms a fluorescent product 2, 7- naphthyridine. JBSNF-000088 was screened using human, mouse and monkey NNMT enzymes. Different concentrations of inhibitors were preincubated along with the enzyme for 30 minutes at room temperature. Reaction was initiated by addition of SAM and nicotinamide mixture (7 µM, 20 µM, 8 µM SAM and 6 µM, 20 µM, 9 µM nicotinamide for human, mouse and monkey NNMT assays respectively) for 60 minutes at 37 °C. The final assay reaction mixture contained a buffer of 100 mM Tris Hcl pH 7.5, 0.04% BSA, 2 mM dithiothreitol and 1% DMSO. At the end of the incubation, the reaction was stopped by the addition of ethanol: acetophenone mix (75% ethanol: 25% acetophenone) and 5 M potassium hydroxide prepared with 50% ethanol. The reaction was incubated for 15 minutes following which 100 µL of 60% formic acid was added. The reaction was incubated for another 60 minutes at room temperature. The fluorescent product 2, 7- naphthyridine was measured using a Tecan reader with excitation at 375 nm and emission at 430 nm. The IC_50_ values were determined by fitting the inhibition curves (percent inhibition versus inhibitor concentration) using a four parametric sigmoidal dose response using GraphPad Prism software.

### Bio-analytical method for the detection of MNA for ***in-vitro*** assays

Samples from both biochemical and cell based assays were analyzed for MNA employing a fit-for-purpose LC-MS/MS method. PE-Sciex API-4000 triple quadrupole (PerkinElmerSciex Instruments, Boston, MA) mass spectrometer was employed for this analysis. Samples (5 µL) were injected onto Atlantis dC18 (50 × 4.6 mm, 3 µm; Waters, Milford, MA, USA) column connected to Shimadzu VP (Shimadzu, Japan) LC system. The isocratic mobile phase, a mixture of 0.2% formic acid and acetonitrile mixture (10:90, v/v) was filtered through a 0.45 μm membrane filter (XI5522050) (Millipore, USA or equivalent) and then degassed ultrasonically for 5 minutes was delivered at a flow rate of 0.65 mL/min into the mass spectrometer electrospray ionization (ESI) chamber. Quantitation was achieved by MS/MS detection in positive ion mode for MNA and internal standard (d4 –MNA). Detection of the ions was performed in the multiple reaction monitoring (MRM) mode, monitoring the transition of the m/z 137 precursor ion to the m/z 92 product ion for MNA and m/z 141 precursor ion to the m/z 84 product ion for d4-MNA (internal standard). The retention times of MNA and d4-MNA were 1.31 minutes and 1.30 minutes, respectively.

### Sample preparation for ***in-vitro*** assays

A simple protein precipitation extraction method was followed for extraction of N-methylnicotinamide from cellular and biochemical cell lysate samples. To an aliquot of ~100 μL of cell lysate samples ~300 µL of acetonitrile containing IS (d4-MNA) solution was added, followed by vortex for ~3 minutes and centrifuged for 10 minutes at 14,000 rpm. The supernatant was separated and transferred to pre-labeled HPLC vials and analyzed by LC-MS/MS system.

### Human NNMT enzymatic assay using LC-MS/MS detection

MNA formed in the human NNMT reaction was measured using a fit-for-purpose LC-MS/MS method. Different concentrations of the inhibitors were preincubated along with 5ng/well human NNMT enzyme for 30 minutes at room temperature. Reaction was initiated by addition of SAM and nicotinamide mixture at 7 µM, 20 µM respectively and incubated for 60 minutes at 37 °C. The final assay reaction mixture contained a buffer of 100 mM Tris Hcl pH 7.5, 0.04% BSA, 2 mM dithiothreitol and 1% DMSO. 100 µL of acetonitrile containing internal standard d_4_-MNA (20ng/mL) was added to the wells and incubated for 10 minutes at room temperature. 70 µL of autoclaved water was added to the wells and mixed gently. The plate was centrifuged at 5000 g for 10 minutes at room temperature. 150 µL of supernatant was transferred into 96 well plate (Costar 3364) and analyzed by LC-MS/MS. The IC_50_ values were determined by fitting the inhibition curves (percent inhibition versus inhibitor concentration) using a four parametric sigmoidal dose response graph pad prism.

### Cell Based U2OS Assay

Human bone osteosarcoma (U2OS) cell line was procured from ATCC and maintained in DMEM F-12 growth media containing 10% heat inactivated fetal bovine serum and penstrep (filter sterilized) at 37 °C with 5% CO2. Cells were counted and 10 K cells per well were seeded into a 96 well cell culture plate followed by incubation for 24 h at 37 °C, 5% CO2, and 95% humidity. Cell culture media was replaced with 100 µl of medium/ inhibitor mixture at different concentrations and incubated for 24 hours at 37 °C, 5% CO2, and 95% humidity. Medium/compound mixture was removed and washed twice followed by addition of 100 µL Acetonitrile containing internal standard d_4_-MNA (20ng/mL) to the wells. The plate was centrifuged at 5000 g for 10 minutes. 150 µL of supernatant was transferred into 96 well plate (Costar 3364) and analyzed by LC-MS/MS. The IC_50_ values were determined by fitting the inhibition curves (percent inhibition versus inhibitor concentration) using a four parametric sigmoidal dose response graph pad prism.

### Cell Based 3T3L1 Assay

3T3-L1, a cell line derived from mouse 3T3 cells was procured from ATCC and maintained in DMEM high glucose growth media containing 10% heat inactivated fetal bovine serum and penstrep at 37 °C with 5% CO2. Cells were counted and 5 K cells per well were seeded into a 96 well cell culture plate and incubated at 37 °C, 5% CO2, 95% humidity until it reached 100% confluency. Post 24 h of confluency the medium was replaced with differentiation induction media containing (500 μM, IBMX + 1 μM DEXA + 1 μg/ml insulin) and the medium was changed on alternate days. Cells were differentiated for up to 14 days: After three days, the induction medium was replaced by DMEM supplemented with 10% FBS and 1 µg/ml insulin. From day 5, cells were cultured in regular growth medium.

Post differentiation, cells were incubated with compound for 24 h at 37 °C, 5% CO2, and 95% humidity. Cells were washed with DPBS and 100 µl acetonitrile containing internal standard d4-MNA (20ng/mL final concentration) was added to the wells. 100% Acetonitrile was used to extract MNA from the cells. The plate was incubated for 20 minutes at room temperature and 100 µL of autoclaved water was added and mixed gently. The plate was centrifuged at 5000 g for 10 minutes. Supernatant was transferred into 96 well plate (Costar 3364) and submitted for LCMS/MS. The IC_50_ values were determined by fitting the inhibition curves (percent inhibition versus inhibitor concentration) using a four parametric sigmoidal dose response graph pad prism.

### Cytotoxicity assay

Human liver cancer (HepG2) cell line was procured from ATCC and maintained in DMEM Glut Max growth media containing 10% HI FBS and 1% Pen-Strep (filter sterilized) in 37 °C incubator with 5% CO_2_. Cells were detached with 0.25% trypsin (Sigma) when they were 80% confluent in a cell culture flask. Cells were counted and 50 K cells per well were seeded into a 96 well opaque-walled multi well plate followed by Incubation overnight at 37 °C, 5% CO_2_, 95% humidity. Cell culture media was replaced with 100 µL of medium/ inhibitor mixture (Containing 0.5% DMSO) along with controls and incubated for 48 or 72 h at 37 °C, 5% CO_2_, 95% humidity. Max control samples (complete reaction with 0.5% DMSO) and min control samples (complete reaction with known inhibitor) Cell Titer-Glo® Reagent (Promega) was added to all the wells containing media in 1:1 ratio (e.g., add 100 µL of reagent to 100 µL of medium containing cells for a 96-well plate). The contents were mixed for 2 minutes on an orbital shaker to induce cell lysis. Then the plate was incubated at room temperature for 10 minutes to stabilize luminescent signal. Luminescence was recorded in Victor or Top Count Luminescence counter. The cell viability over DMSO control was calculated.

### Protein Sample Preparation for Assay

The full length NNMT proteins (1–264 amino acids) of human & mouse (UniProt accession # P40261 & O55239) with an amino terminal 6 × His tag and TEV cleavage site (ENLYFQG) were cloned into pET23a between NdeI and XhoI restriction sites and expressed in *E. coli* Rosetta2(DE3)pLysS cells. The recombinant proteins were purified as described previously^24^.

### Protein Sample Preparation for Structure

The DNA fragment of human & mouse NNMT comprising amino acids 1–264 were cloned into pET28a expression vector between *NdeI* and *XhoI* restriction sites. Three point mutations were introduced in human NNMT (K100A/E101A/E103A) using the QuikChange kit (Stratagene). The proteins were produced and purified from *E. coli* as previously described^24^. Final purified human NNMT sample was concentrated to 12 mg/mL in buffer (20 mM HEPES pH 7.5, 50 mM NaCl, 5% (v/v) glycerol, 1 mM TCEP) and mouse NNMT sample was concentrated to 12 mg/mL in buffer (20 mM HEPES pH 7.5, 125 mM NaCl, 5% (v/v) glycerol, 1 mM TCEP)

### Crystallization and data collection

Small crystal hit for human NMMT complex obtained under the condition containing 0.2 M NaCl, 0.1 M BIS-TRIS pH 5.8, 25%(w/v) PEG 3350. Better diffracting crystals were obtained in 1–2 weeks after seeding. Crystal hits for mouse NNMT complex obtained under the condition 0.35 M sodium thiocyanate, 20% PEG 3350. Crystals grew in a week’s time and got improved upon using 0.01 M TMAO as additive. Crystals were cryo protected in mother liquor supplemented with the addition of 18–20% (v/v) glycerol. X-ray diffraction data were collected at 100 K from MX2 beam lines of the Australian Synchrotron.

### Structure determination and refinement

Data were processed in P2_1_ and P2_1_2_1_2_1_ space group for human and mouse NNMT respectively. Structure solutions were obtained for both using MOLREP program of CCP4 Suite version 5.0.2^25^ with 3ROD as the start model. Model correction and inspection of electron density map were done using COOT (v 0.5.2)^26^, refinement with REFMAC5 program^27^ and Ligand fitted using AFITT (v1.3.2)^28^ program. R-factors for human NNMT & mouse NNMT complexes converged to R_work_(%)/R_free_(%) 22.4/28.6 and 20.7 / 23.7 respectively. Ramachandran plot calculated using PROCHECK^29^ confirmed good stereochemistry. The atomic coordinates and structure factors for human NNMT – JBSNF-000088 and mouse NNMT – JBSNF-000088 complexes have been deposited in the Protein Data Bank with the accession numbers 5YJF and 5YJI respectively. All the crystal structure figures were generated using PyMOL^30^.

### Thermal Shift Assay

Thermal shift assay experiments were carried out as described previously^24^.

### Animal Studies

#### N-Methylnicotinamide (MNA) bioanalytical method for *in-vivo* samples

Samples for MNA were analyzed employing a fit-for-purpose LC-MS/MS method. PE-Sciex API-4000 triple quadrupole (PerkinElmerSciex Instruments, Boston, MA) mass spectrometer was employed for this analysis. Samples (5 µL) were injected onto Kinetex HILIC (50 × 4.6 mm, 2.6 µm; Phenomenex, Torrance, CA, USA) column connected to Shimadzu VP (Shimadzu, Japan) LC system. The gradient mobile phase mixture of 0.2% formic acid and acetonitrile was delivered at a flow rate of 0.45 ml min-1 into the mass spectrometer electrospray ionization (ESI) chamber. Quantitation was achieved by MS/MS detection in positive ion mode for MNA and internal standard (d4–MNA). Detection of the ions was performed in the multiple reaction monitoring (MRM) mode, monitoring the transition of the m/z 137 precursor ion to the m/z 92 product ion for MNA and m/z 141 precursor ion to the m/z 84 product ion for d4-MNA (internal standard). The retention times of MNA and d_4_-MNA were 2.92 minutes and 2.91 minutes, respectively.

#### Sample preparation

A simple protein precipitation extraction method was followed for extraction of N-methylnicotinamide from plasma and various tissue homogenate samples. The tissue samples were homogenized with 1:3 proportions of phosphate buffer saline (PBS) and this homogenate was used for N-Methylnicotinamide determinations. To an aliquot of ~50 μL of plasma/ liver homogenate/ adipose tissue homogenate samples ~300 µl of acetonitrile containing IS (d_4_-MNA) solution was added, followed by vortex for ~3 minutes and centrifuged for 10 minutes at 14,000 rpm. The supernatant was separated and transferred to pre-labeled HPLC vials and analyzed by LC-MS/MS system.

#### MethylatedJBSNF-000088 (compound 20) bioanalytical method details for *in-vivo* samples

Samples for methylated JBSNF-000088 were analyzed employing a fit-for-purpose LC-MS/MS method. PE-Sciex API-5500 triple quadrupole (PerkinElmerSciex Instruments, Boston, MA) mass spectrometer was employed for this analysis. Samples (2 µL) were injected onto Atlantis dC18 (50 × 4.6 mm, 3 µm; Waters, Milford, MA, USA) column connected to Shimadzu VP (Shimadzu, Japan) LC system. The gradient mobile phase mixture of 0.2% formic acid and acetonitrile was delivered at a flow rate of 0.9 mL min-1 into the mass spectrometer electrospray ionization (ESI) chamber. Quantitation was achieved by MS/MS detection in positive ion mode for methylated JBSNF-000088 and internal standard (Tolbutamide). Detection of the ions was performed in the multiple reaction monitoring (MRM) mode, monitoring the transition of the m/z 168 precursor ion to the m/z 128.8, product ion for MNA and m/z 271 precursor ion to the m/z 91 product ion for tolbutamide (internal standard). The retention times of MNA and tolbutamide were 1.45 minutes and 3.09 minutes, respectively.

#### Formulation preparation

Required quantity of test items was weighed and transferred into a mortar. Test compounds and measured volume of Tween 80 was mixed thoroughly using appropriate method. Small volume of 0.5% hydroxyethylcellulose (HEC) was added to the above under constant mixing. The content was transferred into polypropylene tube. The mortar was rinsed with small volume of 0.5% HEC and the contents transferred into the same polypropylene tube. Finally the volume was made up with 0.5% HEC under constant stirring.

#### Animals

Male C57BL6/N (Vivo Bio Tech Ltd, India), ob/ob (B6.V-Lepob/OlaHsd) and its lean control (Envigo laboratories Inc, USA) and db/db (BKS.Cg- + Leprdb/ + Leprdb/OlaHsd) and its lean control (Envigo laboratories Inc, USA) were housed in individually ventilated cages (n = 5/cage) under standard laboratory conditions. The study room environment was maintained at a temperature of 21–24 °C and, relative humidity at 40–70%. The animal rooms were maintained under 12 h light and dark cycle. Animals were provided with *ad libitum* access to water and food (except where noted otherwise, e.g. during fasting prior for glucose tolerance tests, fasting prior to terminal sacrifice). Animals were acclimatized for 1 week before initiation of studies. For Diet induced obesity (DIO) mice study, C57BL6/N mice were maintained on rodents chow diet (Teklad; Harlan) or HFD (60% Kcal; Research diet) for 14 weeks before study initiation and throughout the study period. ob/ob and db/db mice and their lean controls were maintained on rodents chow diet (Teklad; Harlan). All the experiments were approved by Institutional Animal Ethics Committee (IAEC) of Jubilant Biosys Ltd, India.

#### Target Engagement study

Male C57BL6/N mice (Vivo Bio Tech Ltd, India) were used for the target engagement study. On the study day, 24 animals were dosed with JBSNF-000088, 50 mg kg^−1^, p.o. and at every time point 3 animals were bled and sacrificed. Animals were bled and sacrificed after 0.25, 0.5, 1, 2, 4, 6, 8 and 24 h of dosing. 3 animals were bled and sacrificed at 0 minutes (no treatment). Plasma was harvested by centrifuging the blood and stored frozen at −80 ± 10 °C till analysis.

#### Efficacy studies

Lean control + Vehicle and HFD + Vehicle Control groups (G1 and G2) mice were administered with vehicle. Dose formulations of HFD + JBSNF-000088, 50 mg kg^−1^, po, bid were administered at dose volume of 5 mL kg^−1^ body weight to G3 mice. Similar pattern was followed for ob/ob mice and db/db mice studies. The dose volume for individual animals was calculated based on the most recently recorded body weight during the study period. Throughout the study period, all animals were observed for mortality/morbidity. Cage side observations of animals for visible clinical signs, was carried out once daily throughout the study period. Individual animal body weights were recorded twice weekly during the study period.

The ob/ob mice were 12 weeks of age at the start of the study and db/db mice were 8 weeks of age at the start of the study. DIO mice were 20 weeks of age at the start of the study. In each study, non-diabetic lean mice comprised the control group which received vehicle (0.5% w/v HEC and 0.5% v/v Tween 80) and obese or diabetic mice were randomly assigned to two groups based on body weight and unfasted glucose which received either vehicle (0.5% w/v HEC and 0.5% v/v Tween 80) or JBSNF-000088, 50 mg kg^−1^, po, bid for 30 days. Individual animal body weights, food and water consumption were recorded twice weekly during the study period. Each group consisted of 10 animals in DIO, ob/ob and db/db efficacy studies. Animals were housed as n = 5 per cage and individual animal body weight, food and water consumption were recorded twice weekly for the duration of the study. Food consumption was expressed as cumulative energy intake. Fed blood glucose and insulin were measured on day 7, 14 and day 21 post treatment.

#### OGTT

Effect of JBSNF-000088 on glucose tolerance was assessed in an oral glucose tolerance test (OGTT) on day 28 of treatment, in DIO mice, ob/ob mice and on day 26 of treatment for db/db mice. Animals from DIO and ob/ob study were fasted for 4 h followed by an oral administration of glucose (2 g kg^−1^) while animals from db/db study were fasted for 16 h followed by an oral administration of glucose (1 g kg^−1^). One hour prior to glucose administration, mice were dosed orally with vehicle or JBSNF-000088, 50 mg kg^−1^.

Blood glucose measurements from tail snips were performed at −60 (prior to drug administration), 0 (prior to glucose administration), and 15, 30, 60, 120 and 180 minutes after glucose administration. Blood for plasma insulin measurement was collected at 0 and 15 minutes. HOMA-IR was calculated according to the formula; [HOMA-IR = (Fasting plasma insulin, ngml^−1^ × Fasting Blood Glucose, mmol l^−1^) / 22.5]. Animals were re-fed after the last time point of blood glucose and dosing was continued until termination on day 30.

#### Study termination

On day 30 of treatment, animals were fasted for 4 h and sacrificed by CO_2_ asphyxiation. One h prior to sacrifice, mice were dosed orally with vehicle or JBSNF-000088, 50 mg kg^−1^. Blood and tissue samples (liver, subcutaneous fat, renal fat, epididymal fat and mesentery fat) were collected from each animal. Plasma and tissue samples were stored at −80 °C until analysis.

#### Biochemical analysis

Plasma was collected from blood by centrifugation. Plasma insulin levels were determined using an ELISA kit (Mercodia AB, Uppsala, Sweden) as per kit insert. Plasma TG, TC, HDL and LDL were determined by colorimetric methods using commercially available Randox assay kits (Randox Lab., Ltd, UK). Whole blood glucose was estimated using Glucometer (Contur, Bayer)

#### Statistical analysis

Data are reported as the mean ± SEM throughout, and a P value of less than 0.05 was used as the threshold for statistical significance. All statistical analysis was performed using GraphPad Prism-5 software. Two way ANOVA followed by Bonferroni post test was used for Change in body weight, food consumption, weekly fed blood glucose, weekly fed plasma insulin and OGTT. One way ANOVA followed by Bonferroni post test was used to analyze AUC blood glucose in OGTT, 4 h fasting blood glucose, 0 & 15 minutes plasma insulin, HOMA-IR index, MNA in plasma, MNA in liver, MNA in visceral WAT and MNA in S/C WAT.

#### DIO studies using WT and NNMT KO animals

NNMT fl/fl mice on a C57BL/6 background were generated by Regeneron Pharmaceuticals (Tarrytown, NY) using VelociGene® technology^31^. A loxP locus was placed 1.6 kb upstream of the transcription starting site encompassing the gene promoter. A loxP-Frt-Hyg-Frt cassette was inserted 212 bp downstream of NNMT exon 1. The floxed coordinates were chr9:48,412,881–48,414,698. NNMT fl/fl mice were crossed with ZP3-cre mice (C57BL/6, Jackson Laboratories) to generate whole-body NNMT knockout mice. The DIO mouse study was conducted in accordance to the German Animal Protection Law, as well as according to international animal welfare legislation and rules. Female wild-type and NNMT knockout animals were put on high-fat diet (Ssniff HFD adjusted TD.97366) for 18 weeks and then treated with either JBSNF-000088 (50 mg/kg bid by oral gavage) or vehicle for four weeks (n = 9–10 per group). Throughout the study, Mice were housed in an environmentally controlled room at 23 °C on a 12 h:12 h light dark cycle (light on at 06:00 AM), and food and water was offered ad libitum. An oral glucose tolerance test was performed on day 25 of treatment: Animals were fasted overnight. 60 minutes before an oral glucose bolus (2 g kg^−1^), JBSNF-000088 (50 mg kg^−1^) or vehicle (0.5% HEC + 0.5% Tween80) was administered by oral gavage. For blood glucose analysis, blood was collected from the tail of conscious mice at time points 15, 30, 60 and 120 minutes after the glucose bolus.

#### Note added in proof

While this manuscript was being reviewed, Neelakantan *et al*.^32^ published a report describing N-methylquinolines as small-molecule NNMT inhibitors. They showed that 5-Amino-1-methylquinoline causes weight loss at preserved food intake in mice on a high-fat diet, even though the treatment period of 12 days was comparably short. Thus, our results provide independent confirmation of their findings with a structurally different compound and add important information on the effect of NNMT inhibition on insulin sensitivity and glucose tolerance.

### Data availability statement

The datasets generated during and/or analyzed during the current study are available from the corresponding author on reasonable request. Crystal structure coordinates have been deposited at the PDB (accession numbers 5YJF and 5YJI).

## Electronic supplementary material

Supplementary Information
