# Supplementary material for: A small molecule inhibitor of Nicotinamide N-methyltransferase for the treatment of metabolic disorders
Source: Sci Rep. 2018 Feb 26;8:3660. doi: 10.1038/s41598-018-22081-7 (PMC5826917; doi:10.1038/s41598-018-22081-7)
Supplement: Supplementary file 1 — Supplementary Information [file 41598_2018_22081_MOESM1_ESM.docx]

**Supplementary Information to**

**“A small molecule inhibitor of Nicotinamide N-methyltransferase for the treatment of metabolic disorders”**

**By**

Aimo Kannt^1,3^*, Sridharan Rajagopal^2^, Sanjay Venkatachalapathi Kadnur^2^, Juluri Suresh^2^, Ravi Kanth Bhamidipati^2^, Srinivasan Swaminathan^2^, Mahanandeesha Siddappa Hallur^2^, Rajendra Kristam^2^, Ralf Elvert^1^, Jörg Czech^1^, Anja Pfenninger^1^, Christine Rudolph^1^, Herman Schreuder^1^, Devaraj Venkatapura Chandrasekar^2^, Vishal Subhash Mane^2^, Swarnakumari Birudukota^2^, Shama Shaik^2^, Bharat Ravindra Zope^2^, Raghunadha Reddy Burri^2^, Niranjan Naranapura Anand^2^, Manish Kumar Thakur^2^, Manvi Singh^2^, Reejuana Parveen^2^, Saravanan Kandan^2^, Ramesh Mullangi^2^, Takeshi Yura^2^, Ramachandraiah Gosu^2^, Sven Ruf^1^, and Saravanakumar Dhakshinamoorthy^2^*

*^1^Sanofi Research and Development, Industriepark Hoechst, H823, D-65926 Frankfurt am Main, Germany*

*^2^ Jubilant Biosys Ltd, Bangalore-560022, India*

*^3^Institute of Experimental Pharmacology, Medical Faculty Mannheim, University of Heidelberg, D-68167 Mannheim, Germany*

*^*^corresponding authors*

**Supplementary table 1: X-ray data collection and refinement statistics**

| **Crystal Structure** | **Human NNMT+ JBSNF-000088** | **Mouse NNMT+ JBSNF-000088** |
| --- | --- | --- |
| PDB id | 5YJF | 5YJI |
| Space group | P2_1_ | P2_1_2_1_2_1_ |
| Unit Cell dimensions |  |  |
| *a*, *b*, *c* (Å) | 60.32, 132.98, 61.32 | 47.36, 71.62, 157.40 |
| α, β, γ (°) | 90.0, 103.57, 90.0 | 90.0, 90.0, 90.0 |
| **Data collection** |  |  |
| Source | Australian Synchrotron  (MX2 Beamlines) | Australian Synchrotron  (MX2 Beamlines) |
| Wavelength (Å) | 0.95370 | 0.95370 |
| Resolution range (Å) | 47.78 – 2.48 (2.58 – 2.48) | 47.36 -1.99 (2.03 – 1.99) |
| Observed Reflections | 205585 | 359287 |
| Unique reflections | 31948 | 37822 |
| Completeness (%) | 96.4 (86.4) | 99.6 (95.2) |
| Multiplicity | 6.4 (5.0) | 9.5 (8.5) |
| *<I>*/ σ <*I>)* | 5.7 (1.3) | 14.2 (4.3) |
| $\dagger$*R*_merge_ (%) | 19.2 (88.5) | 12.6 (52.0) |
| ††*R*_pim_ (%) | 8.0 (41.1) | 4.1(18.2) |
| **Refinement** |  |  |
| Resolution range (Å) | 47.78 - 2.48 | 47.36 - 1.99 |
| No of reflections in |  |  |
| Working set | 30164 | 35850 |
| Test set | 1617 | 1890 |
| Wilson B-factor | 41.5 |  |
| ‡*R*_work_ / §*R*_free_ | 0.224 / 0.286 | 0.210 / 0.238 |
| No of atoms | 8241 | 4350 |
| Macromolecules | 8018 | 4063 |
| Ligand | 152 | 76 |
| Water | 71 | 211 |
| No of residues / Total residues | A chain = 256 / 284  B chain = 256 / 284  C chain = 257 / 284  D chain = 252 / 284 | A chain = 256 / 284  B chain = 252 / 284 |
| Missing residues | 115 | 60 |
| R.m.s deviations |  |  |
| Bond lengths (Å) | 0.009 | 0.008 |
| Bond angles (°) | 1.294 | 1.153 |
| Ramachandran analysis |  |  |
| Core (%) | 91.8 | 94.1 |
| Allowed (%) | 8.1 | 5.6 |
| Gen allowed (%) | 0.1 | 0.3 |
| Outliers (%) | 0 | 0 |
| **Average B-factors (Å2)** |  |  |
| Protein chains | A chain = 29.3  B chain = 30.3  C chain = 38.1  D chain = 38.7 | A chain = 16.4  B chain = 22.9 |
| Ligand/ion | 29.1 / 27.2 | 11.5 / 14.3 |
| Water | 26.4 | 25.8 |

$\dagger Rmerge=\Sigma|I-<I>|/\Sigma I\times100,$ where *I* is intensity of a reflection and $<I>$ is its average intensity

$$\dagger\dagger Rpim=\Sigma\surd(\frac{1}{N-1})\times\Sigma|I-<I>|/\Sigma I\times100$$

$$\ddagger Rwork=\Sigma\left| \mathrm{Fo}-\mathrm{Fc} \right|/\Sigma|\mathrm{Fo}|\times100$$

§ Rfree is calculated on 5% randomly selected reflections, for cross-validation. Values in parentheses represent the highest resolution shell.

**Supplementary table 2: Selectivity profile of JBSNF-000088 (at 10 µM) vs a panel of 34 receptors, channels and enzymes.**

|  | E% (agonistic) | I% (antagonistic or inhibitory) |
| --- | --- | --- |
| cGMP-inhibited 3,5-cyclic phosphodiesterase A (PDEA3) |  | -1 |
| Acetylcholinesterase (AChE) |  | 2 |
| Adenosine Receptor 1 (ADORA1) | -3 | -1 |
| Adenosine Receptor 2A (ADORA2A) | 4 | 2 |
| adrenergic, alpha-1A-, receptor (ADRA1A) | -2 | -3 |
| Adrenoreceptor alpha 2A (ADRA2A) | 4 | -12 |
| Adrenoreceptor beta 1 (ADRB1) | 3 | -33 |
| Adrenoreceptor beta 2 (ADRB2) | 1 | 1 |
| Ca2+ Channel (L-Type DHP Site) |  | 0 |
| Cannabinoid Receptor 1 (CB1) | 0 | 1 |
| Cannabinoid Receptor 2 (CB2) | -7 | 1 |
| Cl- Channel |  | 8 |
| Dopamine Receptor 1 (D1) | 0 | -3 |
| Dopamine Receptor 2 Short (D2S) | -3 | 12 |
| Dopamine Transporter |  | 7 |
| Glycine Receptor (strychnine-sensitive) |  | -7 |
| Histamine Receptor 1 (H1) | -2 | -5 |
| Histamine Receptor 2 (H2) | -1 | 6 |
| K+ Channel Voltage Dependent |  | 3 |
| Mono Amine Oxydase A (MAO-A) |  | -1 |
| Muscarinic Receptor 1 (CHRM1) | -3 | -7 |
| Muscarinic Receptor 2 (CHRM2) | 13 | 3 |
| Muscarinic Receptor 3 (CHRM3) | -1 | 21 |
| Na+/K+ ATPase Pump |  | 0 |
| Niacin receptor (NAR1) | -3 |  |
| Nicotinic AChR alpha4 beta2 (nAChRa4b2) |  | 5 |
| Nicotinic AChR Receptor Muscle-Type |  | -2 |
| Norepinephrin (Noradrenalin) Transporter |  | 9 |
| Opioid Receptor mu (OPRM1) | -1 | -8 |
| Phencyclidine Receptor (PCP) |  | -11 |
| Serotonin Receptor 1A (5HT1A) | 5 | -13 |
| Serotonin Receptor 2A (5HT2A) | 3 | -9 |
| Serotonin receptor 2B (5HT2B | 4 | 13 |
| SK+Ca channel- |  | -4 |
